# Integrating HIV, syphilis, malaria and anaemia point-of-care testing (POCT) for antenatal care at dispensaries in western Kenya: discrete-event simulation modelling of operational impact

**DOI:** 10.1186/s12889-019-7739-4

**Published:** 2019-12-03

**Authors:** N. Young, M. Taetgmeyer, G. Zulaika, G. Aol, M. Desai, F. Ter Kuile, I. Langley

**Affiliations:** 1 Department of International Public Health, Liverpool School of Tropical Medicine, Liverpool, UK; 2 Department of Clinical Sciences, Liverpool School of Tropical Medicine, Liverpool, UK; 3 Tropical Infectious Disease Unit, Royal Liverpool University Hospital, Liverpool, UK; 4 Kenya Medical Research Institute, Center for Global Health Research, Kisumu, Kenya; 5 Malaria Branch, Division of Parasitic Diseases and Malaria, Center for Global Health, Centers for Disease Control and Prevention, Atlanta, GA USA

**Keywords:** Antenatal care, Kenya, Discrete-event simulation modelling, Integration, Point-of-care testing, HIV, Syphilis, Malaria, Anaemia, Human resources

## Abstract

**Background:**

Despite WHO advocating for an integrated approach to antenatal care (ANC), testing coverage for conditions other than HIV remains low and women are referred to distant laboratories for testing. Using point-of-care tests (POCTs) at peripheral dispensaries could improve access to testing and timely treatment. However, the effect of providing additional services on nurse workload and client wait times are unknown. We use discrete-event simulation (DES) modelling to understand the effect of providing four point-of-care tests for ANC on nurse utilization and wait times for women seeking maternal and child health (MCH) services.

**Methods:**

We collected detailed time-motion data over 20 days from one high volume dispensary in western Kenya during the 8-month implementation period (2014–2015) of the intervention. We constructed a simulation model using empirical arrival distributions, activity durations and client pathways of women seeking MCH services. We removed the intervention from the model to obtain wait times, length-of-stay and nurse utilization rates for the baseline scenario where only HIV testing was offered for ANC. Additionally, we modelled a scenario where nurse consultations were set to have minimum durations for sufficient delivery of all WHO-recommended services.

**Results:**

A total of 183 women visited the dispensary for MCH services and 14 of these women received point-of-care testing (POCT). The mean difference in total waiting time was 2 min (95%CI: < 1–4 min, *p* = 0.026) for MCH women when integrated POCT was given, and 9 min (95%CI: 4–14 min, *p* < 0.001) when integrated POCT with adequate ANC consult times was given compared to the baseline scenario. Mean length-of-stay increased by 2 min (95%CI: < 1–4 min, *p* = 0.015) with integrated POCT and by 16 min (95%CI: 10–21 min, *p* < 0.001) with integrated POCT and adequate consult times compared to the baseline scenario. The two nurses’ overall daily utilization in the scenario with sufficient minimum consult durations were 72 and 75%.

**Conclusion:**

The intervention had a modest overall impact on wait times and length-of-stay for women seeking MCH services while ensuring pregnant women received essential diagnostic testing. Nurse utilization rates fluctuated among days: nurses experienced spikes in workload on some days but were under-utilized on the majority of days. Overall, our model suggests there was sufficient time to deliver all WHO’s required ANC activities and offer integrated testing for ANC first and re-visits with the current number of healthcare staff. Further investigations on improving healthcare worker, availability, performance and quality of care are needed. Delivering four point-of-care tests together for ANC at dispensary level would be a low burden strategy to improve ANC.

## Background

HIV, syphilis, malaria, and anaemia are leading preventable causes of adverse pregnancy outcomes in sub-Saharan Africa (SSA) and addressing them as early as possible during pregnancy is an essential goal of antenatal care (ANC) [1]. Kenyan guidelines require screening for HIV, syphilis, and anaemia at the first ANC visit [2]. While over 95% of pregnant women receive ANC and over 90% are tested for HIV in Kenya [3], fewer than half are ever tested for syphilis or anaemia during their pregnancy [3–5]. This difference in coverage is partly due to low testing availability at peripheral facilities (dispensaries) [6, 7] where most women seek ANC. Dispensaries, which do not have laboratories, outnumber health centres which have laboratories by three to one. Syphilis and anaemia testing are still regarded as laboratory-based tests and pregnant women who visit dispensaries are referred, with additional time and cost implications, to more distant health centres for testing. International advocacy for HIV has promoted widespread testing coverage [8, 9], even in the hinterlands, and similar support is needed for syphilis and anaemia testing given the strong evidence of their clinical effectiveness in improving pregnancy outcomes [1, 10, 11]. For malaria endemic regions, Kenya currently does not require parasitological screening in pregnancy, but microscopy is commonly done for screening at health centres in western Kenya. It is noteworthy that Kenya’s neighbour, Tanzania, recently introduced malaria testing at first contact for managing anaemia [12]. Furthermore, there is heightened interest in malaria testing and treatment at first contact because of concerns with current preventive strategies including: 1) poor coverage of intermittent preventive therapy with sulfadoxine-pyrimethamine and bed-net use [13], 2) increasing drug resistance [14], and 3) contraindications to the use of sulfadoxine-pyrimethamine in the first trimester of pregnancy and HIV positive women on cotrimoxazole [15]. Integrating malaria testing with other essential testing requires little additional effort because of testing synergies including using blood from one fingerpick to run the tests and the tests having similar run times. *Moreover, parasitaemia is the highest in the first trimester (between 9 and 16 gestation weeks)* [16] *and more likely to be detected with a rapid test.*

An integrated approach where antenatal testing and appropriate treatment are delivered as a one-stop-shop at a single service delivery point is advocated to reduce missed opportunities and improve coverage of interventions [17]. No-equipment rapid diagnostic point-of-care tests (POCTs) are available to fulfil antenatal testing requirements in low-resource settings [18]. Studies that have assessed the use of syphilis or malaria POCTs have reported ease-of-use, increased healthcare workers’ satisfaction, and improved clients’ trust in the diagnoses due to the observability of results [4, 9, 19–22]. While dual HIV/syphilis tests are now available and countries are beginning to adopt them [23], no study to our knowledge has examined the integration of four essential POCTs for ANC at dispensaries.

At the lowest level, dispensaries offer basic maternal and child health services, rudimentary out-patient curative care and support care for HIV-positive clients, and referrals. Staff at peripheral facilities tend to be overburdened [24] and adding new tasks may impose additional time and resource demands on service delivery which can lead to longer wait times, negatively affecting patient experiences and their health seeking behaviour [25, 26]. Despite this, the World Health Report 2010 estimates that 20–40% of health spending is wasted through inefficiency [27] and there is evidence that the existing workforce is not fully utilized [28–30]. Quantifying wait times and staff utilization is important to understand the likely impact of expanding POCT beyond HIV testing alone so strategies can be targeted to improve adoption and quality of care.

Health systems are complex and adaptive. They display emergent behaviour where the collective whole of the system is more complex than the sum of its parts [31]. Complex systems are nonlinear and traditional analytic approaches, such as regression modelling, are limiting because they cannot account for feedback loops and nonlinear dynamics [32]. Changes in operational processes may have knock-on downstream effects and the overall impact on the system cannot be easily predicted. Operational research methods that use advanced mathematical and modelling techniques can be more appropriate to aid complex decision-making [33]. Discrete-event simulation (DES) modelling is particularly useful for quantifying changes in wait times and resource utilization because it captures ‘discrete’ events such as activities along the client pathway and can introduce decision logic at specific points to simulate competition for resources [34]. While DES has been used extensively in developed countries [35] few examples are available from SSA [36–38]. Using DES, we aim to explore the impact of the integrated testing strategy for ANC on women’s wait times, length-of-stay and resource utilization. The results of this study will also demonstrate the applicability of the method for understanding intervention adoption in complex health systems.

## Methods

### Study setting

Modelling was nested within an 8-month longitudinal study (December 2014 to August 2015) that implemented an integrated testing strategy for HIV, syphilis, malaria, and anaemia in seven dispensaries within the Kenya Medical Research Institute (KEMRI) and US Centers for Disease Control and Prevention’s (CDC) Health and Demographic Surveillance System (HDSS) area in Siaya County, western Kenya [7]. At the time of the study, there were 37 public health facilities in the HDSS area: one district hospital, nine health centres and 27 dispensaries. Detailed population characteristics and setting descriptions are available [39]. The Government of Kenya routinely supplied HIV POCTs per its standard national algorithm at the time: HIV (1 + 2) Antibody Colloidal Gold (KHB, Shanghai Kehua Bio-engineering Co Ltd., China) for screening, First Response HIV-1-2 kits (Premier Medical Corporation Ltd., Kachigam, India) for confirmation and Uni-Gold™ (Trinity Biotech, Ireland) for tie-breaking. The study supplied POCTs for syphilis (SD BIOLINE Syphilis 3.0, Standard Diagnostic Inc., Korea), malaria (CareStart™ Malaria HRP2 Pf, AccessBio, USA) and haemoglobin concentrations (HemoCue® Hb 201+, HemoCue AB, Sweden). During implementation, the seven study dispensaries received a monthly median of 38 (IQR: 32–38) antenatal visits, of which a median of 13 (IQR: 10–13) were first visits. Implementation outcomes from the study showed high adoption of POCTs, resulting in increased case detection and 70% treatment fidelity for syphilis and malaria [7].

**Fig. 1:**
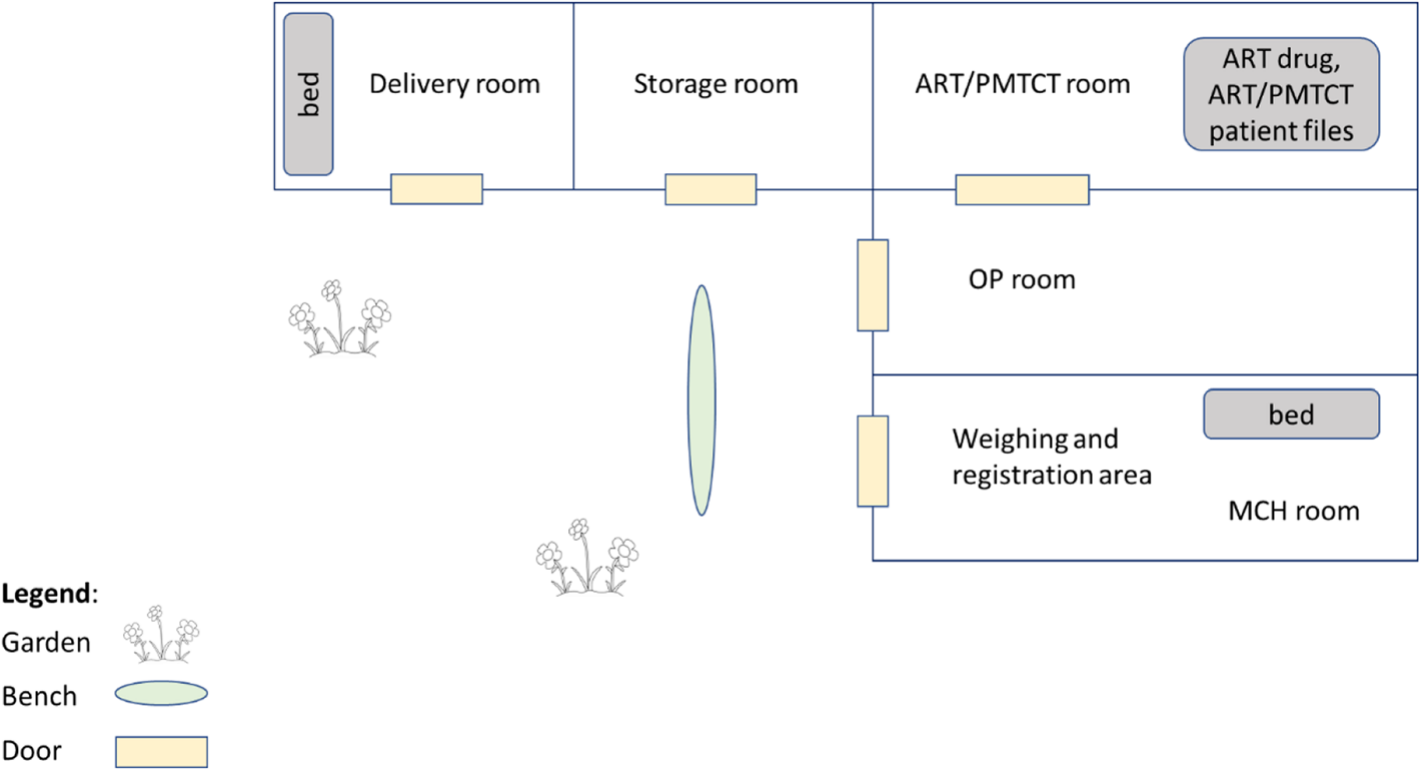
Facility floorplan

Of the seven dispensaries, we conducted our modelling study in one with high client volume. The facility had the typical staffing profile of a dispensary: two nurses, one focused on maternal and child health (MCH) visits, and the other on out-patient (OP) visits; an HIV testing counsellor (HTC) who conducted provider-initiated HIV testing; a part-time clinical officer (CO) who oversaw HIV-positive clients seeking anti-retroviral treatment (ART) or prevention of mother-to-child transmission (PMTCT) services; and two to three subordinate support staff who helped with registration, weighing, and dispensing drugs. The facility had three main rooms, one each for MCH, OP, and ART/PMTCT (Fig. 1). Staff rotated among these rooms for the respective services.

### Time-motion study

We collected time-motion data at the facility over 4 weeks in August 2015 during the intervention. Six data collectors were stationed across each facility service point: two by the entrance, two at the MCH room, one at the OP room, and one at the ART/PMTCT room. Firstly, data collectors recorded all facility clients’ arrival times and purpose of visit (including MCH and non-MCH visit) to inform client mix and arrival time distributions. Secondly, for all MCH visits, we collected detailed information on client pathways. Any woman arriving at the facility for MCH purposes was greeted and introduced to the study. A short statement explained the study purpose (to measure activity and wait times), study procedures (wear a number badge and carry a timesheet throughout the visit), and confidentiality (no personal information such as name or test results would be collected). Women who disagreed would be free to continue their visits without timesheets and their badge numbers would be skipped. Data collectors present at each service point recorded activity process start and end times, service locations, provider type, and any blood tests done on the MCH women’s timesheets. Thirdly, data collectors recorded how healthcare workers (nurses, and COs) were spending their time (e.g. attending to clients, doing paperwork, doing miscellaneous tasks, taking breaks or unavailable) at every 15-min interval from the time of the healthcare workers’ arrival until their departure. All the data were recorded with established reference codes and any unforeseen items were given new codes that were communicated to the team immediately. Digital watches were synchronized daily at the beginning and end. All timesheets were scanned by TeleForm® (Hewlett-Packard) and exported into an excel database.

### Modelling

A deterministic DES model of the dispensary was built in WITNESS© (Lanner Group Limited) simulation software. The model is made of entities, attributes, resources, and activities. Entities are people or items that enter the system (e.g. clients, paperwork) and require attention from resources (e.g. nurses, HTC, CO). The resources attend to entities in activities (e.g. consultation, registration). Attributes are intrinsic features of entities such as client types, pathways and the time spent on activities. Queues are generated in the DES model when entities compete for resources who are often needed in several activities simultaneously. The model outputs include waiting times, length-of-stay and nurse utilizations. We report wait times and length-of-stay in hour and minute format (hh:mm). The total wait time is defined as the sum total of time women had to wait for services and the length-of-stay is the time between women’s arrival and departure.

#### Development and validation of the base-model with integrated POCT

Empirically collected time-motion data was used to inform model inputs. These inputs were: 1) all facility clients’ arrival times which included MCH and non-MCH clients; 2) all MCH clients’ activity sequences, activity durations, activity locations and activity service providers; and 3) non-MCH clients’ OP and ART consultation durations.

All entities entered the model with their empirical arrival times. We made assumptions about OP and ART visits because we did not follow non-MCH clients: we assumed every OP and ART client had a consultation with either a nurse or a CO and that none of them were turned away except for those who came on the day healthcare workers were striking. Their consultation durations were given distributions estimated from those of MCH women who received OP or ART services with an average of 6.5 min for OP consult with nurses, 5 min for ART consult with nurses and 10 min for ART consult with the CO. MCH entities were given their empirical client flow sequences and activity durations as attributes. Healthcare worker availabilities and shift patterns were informed by the healthcare worker activity observations.

Empirical and model-generated distributions of total wait times and length-of-stay for MCH clients were compared for validation.

#### Isolating the impact of integrated POCT

Before the 8-month longitudinal intervention study, only HIV testing was routinely performed at the dispensary. The intervention was defined as the integration of additional syphilis, malaria and anaemia point-of-care testing with routine antenatal HIV testing. As the time-motion data was collected while the intervention was present, we had to generate our primary output distributions without the intervention to create the baseline scenario ([0] without integrated POCT). We did this by removing the process durations of the additional testing from the empirical data. The time needed for the additional testing was estimated to be 8 min: 3 additional minutes for preparing the syphilis, malaria, and anaemia tests and 5 extra minutes to read the syphilis and malaria test results (HIV test requires 15 min for a negative reading while the syphilis and malaria tests require 20 min).

#### ‘What-if’ scenario

We explored a ‘what-if’ scenario where all ANC consultations were of sufficient *minimum* durations to cover all recommended services, including integrated testing. Using data from client-provider role-play interactions in Tanzania [40], we estimated that a minimum of 58 and 36 min would be required to cover all recommended services in first visit and re-visit ANC consultations respectively (Table 1). ANC consultations that were shorter than the minimum durations were increased to their minimum values while those that were longer than the minimum durations retained their empirical values.

**Table 1:** Estimated ideal times for antenatal first visit and antenatal revisit based on consultation times estimated from Tanzania in hours and minutes (hh:mm) [40]

Activity	First visit	Revisit
Welcoming the woman	00:01	00:01
Registration	00:05	00:00
History taking/updating	00:10	00:05
Physical exam	00:08	00:08
Drug administration	00:03	00:03
Immunization	00:01	00:01
Pre-test counselling and set up	00:05	00:00
Health education and counselling while waiting for POCTs results	00:20	00:15
Post-test results counselling and treatment	00:02	00:00
Filling ANC book and register	00:03	00:03
TOTAL TIME	00:58	00:36

POCTs point-of-care tests, ANC antenatal care

Mean wait times and length-of-stay under the scenarios with integrated POCT [1], and with integrated POCT and adequate consult times [2] were compared with the baseline scenario [0] using paired t-tests.

## Results

### Facility characteristics

**Fig. 2:**
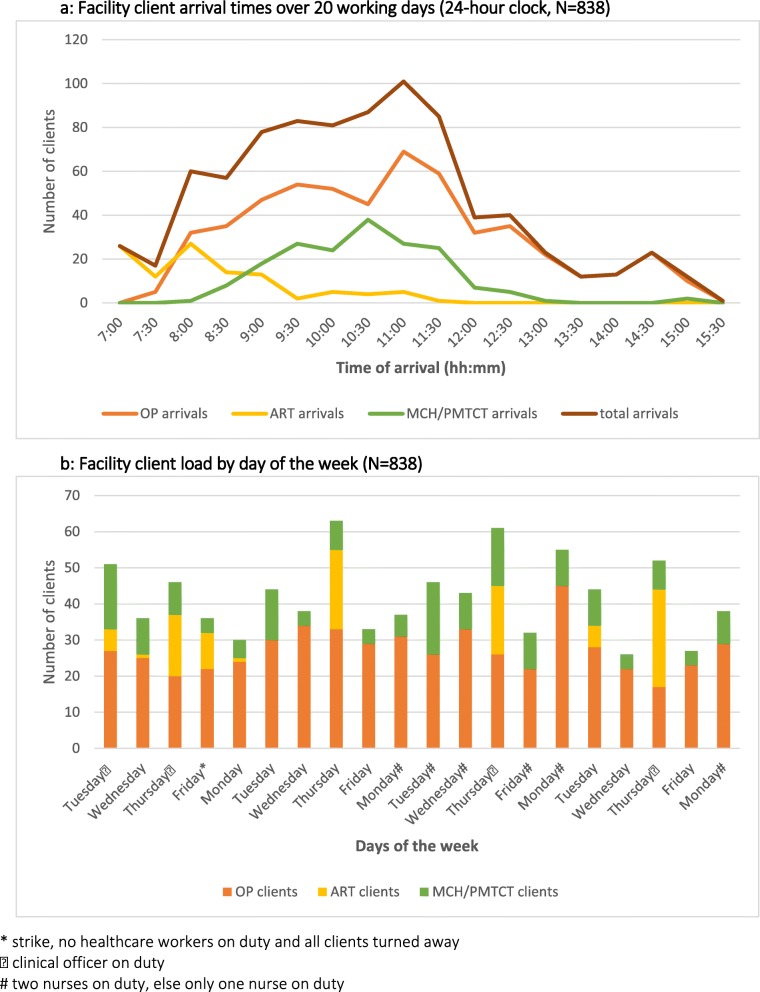
**a**: Facility client arrival times over 20 working days (24-h clock, N= 838). **b:** Facility client load by day of the week (N = 838)

Over 20 days, the facility received 109 (13%) HIV-positive clients for ART, 546 (65%) out-patient clients and 183 (22%) MCH clients. MCH visit purposes included ANC first (n = 12) and re-visits (n = 28), PMTCT (n = 24), family planning (n = 13), under-5 child welfare services for growth monitoring and immunizations (n = 104), labour (n = 1), and pregnancy sick visit (n = 1). All MCH women were approached and all agreed to participate. Figures 2a and b shows the distribution of daily arrival times and client load by day of the week.

Nurses typically arrive at 08:00 and finish their work by 16:00 contributing to an 8-h (480 min) day. The CO was part-time and usually present on Thursdays which is the facility’s designated PMTCT/ART day. Healthcare workers were on strike on the first Friday and no clients were seen that day.

### Model validation

**Fig. 3:**
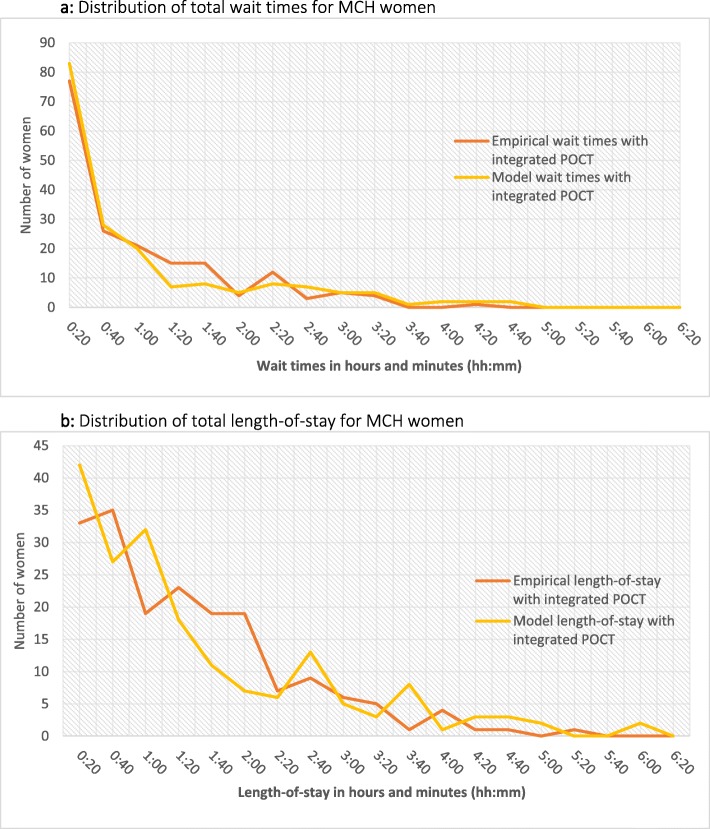
**a**: Distribution of total wait times for MCH women. **b**: Distribution of total length-of-stay for MCH women

Output distribution of wait times and length-of-stay for MCH visits were non-normal even after log and square transformations. Empirical and model generated distributions were compared and shown to be similar (Fig. 3a and b). We concluded that the model was representative of the operational environment of the facility.

### Model generated wait times and length-of-stay

The model was run for the three scenarios with all inputs unchanged except for the activity durations of some of the ANC consultations. Under scenario [1] with integrated POCT, 14 MCH women (11 out of 12 first visits, and three out of 28 re-visits) received the intervention during their nurse consultations (the one first visit woman who did not receive integrated testing was found to be not pregnant after pregnancy testing). First visit consultations took a median of 00:32 (n = 11, range: 00:15–1:14, IQR: 00:23–00:36) and re-visits 00:13 (n = 28, range: 00:05–00:55:00, IQR: 00:09–00:23).

To create the baseline scenario without integrated POCT [0], nurse consultation times were reduced by 8 min for 11 of the 14 women who received the intervention. Consult times were not reduced for three women because they were less than 20 min, the minimum time needed for reading negative results (positive results can be read earlier).

For the ‘what-if’ scenario with integrated POCT and adequate consult times [2], 10 first visit and 23 re-visit ANC consult times were insufficient to cover all required ANC activities and were thus increased to minimums of 58 and 36 min respectively.

Median and mean wait times and length-of-stay under the three scenarios are shown in Table 2. The mean difference in total wait time was + 00:02 (95%CI: 00:00–00:04, *p* = 0.026) for MCH women when integrated POCT was included in the model, and + 00:09 (95%CI: 00:04–00:14, *p* < 0.001) when integrated POCT with adequate ANC consult times were included in the model compared to the baseline scenario. Mean length-of-stay increased by 00:02 (95%CI: 00:00–00:04, 0 = 0.015) with integrated POCT and by 00:16 (95%CI: 00:10–00:21, p < 0.001) with integrated POCT and adequate consult times compared to the baseline scenario. Some women in the upper quartile had very long wait times (over 3 h). These long wait times were the result of having to wait for multiple services from different providers, especially for PMTCT women who had to queue for their MCH consult with the nurse and PMTCT consult with CO.

**Table 2:** Wait times and length-of-stay in hh:mm under the three scenarios with mean differences

	Median (IQR, max)	Mean (95%CI)	Mean difference (95%CI)	*p*-value^a^
Wait times (N = 183)				
[0] Without integrated POCT	00:22 (00:01–01:21, 04:31)	00:50 (00:41–00:59)	reference	
[1] With integrated POCT	00:24 (00:01–01:15, 04:31)	00:52 (00:42–00:61)	00:02 (00:00–00:04)	0.026
[2] With integrated POCT and adequate consult times	00:31 (00:04–01:31, 05:35)	00:59 (00:49–00:69)	00:09 (00:04–00:14)	< 0.001
Length-of-stay (N = 183)				
[0] Without integrated POCT	00:52 (00:23–01:56, 05:54)	01:18 (01:08–01:29)	reference	
[1] With integrated POCT	00:55 (00:22–02:01, 05:54)	01:20 (1:09–01:32)	00:02 (00:00–00:04)	0.015
[2] With integrated POCT and adequate consult times	01:08 (00:31–02:14, 07:03)	01:34 (01:22–01:46)	00:16 (00:10–00:21)	< 0.001

^a^paired t-test; *IQR* inter-quartile range

### Nurse availability and utilizations

The nurse utilization is reported as a percentage of the time they were engaged in activities during their time at the facility. For a 480-min day, 80% utilization would leave 96 min for breaks and travelling between service points. Availability and daily utilization rates, with those above 80% bolded, are shown in Table 3. Increasing the minimal duration of first and re-visit consultations led to higher nurse utilizations but remained under 80% on most days.

**Table 3:** Nurse utilization under 3 scenarios

	Nurse 1 (OP) utilization			Nurse 2 (MCH) utilization		
	Without integrated POCT [0]	With integrated POCT [1]	With integrated POCT & adequate consult times [2]	Without integrated POCT [0]	With integrated POCT [1]	With integrated POCT & adequate consult times [2]
Tuesday^a^	–	–	–	323/480 (67%)	345/480 (72%)	**408/480 (85%)**
Wednesday	–	–	–	232/480 (48.3%)	248/480 (52%)	326/480 (68%)
Thursday^a^	–	–	–	186/480 (38.7%)	186/480 (39%)	227/480 (47%)
Friday	–	–	–	0	0	0
Monday	293/480 (61%)	301/480 (63%)	323/480 (67%)	0	0	0
Tuesday	**246/300 (82%)**	**246/300 (82%)**	**305/300 (102%)**^**b**^	0	0	0
Wednesday	295/480 (61%)	295/480 (61%)	295/480 (61%)	0	0	0
Thursday	**529/570 (93%)**	**529/570 (93%)**	**575/570 (101%)**^**b**^	0	0	0
Friday	378/480 (79%)	378/480 (79%)	378/480 (79%)	0	0	0
Monday	302/480 (63%)	302/480 (63%)	332/480 (69%)	323/480 (67%)	323/480 (67%)	344/480 (72%)
Tuesday	166/480 (35%)	166/480 (35%)	178/480 (37%)	230/360 (64%)	230/360 (64%)	**293/360 (81%)**
Wednesday	**413/480 (86%)**	**413/480 (86%)**	**422/480 (88%)**	293/480 (61%)	293/480 (61%)	**385/480 (80%)**
Thursday^a^	0	0	0	303/480 (63%)	314/480 (65%)	377/480 (78%)
Friday	62/120 (52%)	62/120 (52%)	62/120 (52%)	331/480 (69%)	331/480 (69%)	**424/480 (88%)**
Monday	**431/480 (90%)**	**431/480 (90%)**	**431/480 (90%)**	**383/480 (80%)**	**383/480 (80%)**	**399/480 (83%)**
Tuesday	0	0	0	369/480 (77%)	**383/480 (80%)**	**419/480 (87%)**
Wednesday	0	0	0	141/480 (30%)	141/480 (30%)	141/480 (30%)
Thursday^a^	0	0	0	195/480 (41%)	195/480 (41%)	211/480 (44%)
Friday	0	0	0	301/480 (61%)	301/480 (63%)	301/480 (63%)
Monday	216/360 (60%)	216/360 (60%)	216/360 (60%)	275/360 (77%)	283/360 (79%)	**300/360 (83%)**
OVERALL	3331/4710 (71%)	3339/4710 (71%)	3517/4710 (75%)	3885/6480 (60%)	3956/6480 (61%)	4555/6480 (70%)
p-value ^c^		[1] vs [0] *p* = 0.3173	[2] vs [1] *p* = 0.0148		[1] vs [0] *p* = 0.0258	[2] vs [1] *p* = 0.0008

Nurse 1 spent approximately 870 min (18.5%) and Nurse 2 spent approximately 1410 min (21.8%) of their utilization time on paperwork

*OP* out-patient clients, *MCH* maternal and child health clients, *POCT* point-of-care testing

^a^ Clinical officer on duty- out of 390-min shifts, the clinical officer had less than 50% utilization each day. For scenario 3, the CO was modelled to help with child immunization when he was available

^**b**^ Nurse utilizations generated by the model can be over 100% because the resource is given an allowance to carry on processing any on-going activity after the shift has finished

^c^ Wilcoxon sign-rank test

## Discussion

We integrated point-of-care testing for syphilis, malaria and anaemia with HIV testing to fulfil antenatal testing guidelines at first ANC visits in dispensaries in western Kenya. We captured detailed time-motion data to represent the local environment and used discrete-event simulation modelling to quantify wait times, length-of-stay and nurse utilization of the intervention. This study demonstrated the applicability of simulation modelling to help understand the operational consequences of implementing priority interventions in low-resource settings.

Having nurses deliver integrated POCT during ANC consultations did not result in substantial increases in wait times or length-of-stay. This lack of substantial increase in wait times or length-of-stay was because only a small proportion of MCH women required testing. Moreover, testing procedures were able to synergize with HIV testing to maximize time efficiencies. Qualitative interviews with healthcare workers at facilities implementing the intervention showed they enjoyed providing testing services and found the tests easy to use [41]. Offering POCT testing services at dispensaries such as this one could be a feasible strategy that enables women to be tested at first ANC contact without over-burdening the overall operational environment of the facility. Observed ANC consultations were much shorter than ideal consultation times. This was also reflected in Tanzania where observed ANC consults took an average of 15 min for first visits and 9 min for re-visits [40] and suggests that not all ANC services were given adequately. The gap is likely to be in health education and counselling services as was found in the Tanzanian study [40]. The interviews with healthcare workers implementing POCT suggest that healthcare workers’ frustrations on workload and poor working conditions were felt to compromise quality of care and motivation to give proper counselling and health education [41]. Modelling sufficient time spent for consultations resulted in increase in mean wait times of 9 min (95%CI: 4–14 min) for MCH clients. Further studies on the acceptability and cost-benefit of spending longer times at facilities are needed.

A Canadian study on cardiovascular nursing care in hospitals shows 85% (±5%) daily nurse utilization rate is ideal and sustained utilization above this range can lead to increase in costs, decrease in quality of care as well as poorer nurse and patient outcomes [42]. Our study showed that nurse utilization rates fluctuated across days. On days when client load was high and staffing levels were low, utilization rates reached above 85%. This creates excess workload for the healthcare worker on shift and likely to undermine the quality of care delivered [43, 44]. For 2 days the OP nurse reached 100% utilization under scenario [2]: one was because the OP nurse was alone and only present part-time (300 min) and the other was because the OP nurse was alone on a Thursday when PMTCT/ART clients were scheduled. Absenteeism, whether planned or unplanned, is characteristic of facilities in low-resource settings [43]. Staff are often pulled out of facilities to attend disease-specific trainings, or participate in outreach campaigns [24]. Reducing parallel programmes, integrating training, building on synergies among disease programmes, ensuring appropriate skills are covered in pre-service curricula, and conducting on-site trainings when possible can reduce disruptions [45]. Better scheduling of client visits and ensuring healthcare worker availability on busy days may alleviate these spikes in workload. On the other hand, healthcare workers may feel poorly motivated to show up because of low morale from meagre and delayed salaries, lack of choice in placement, workload, poor working conditions, job grade stagnation, and feeling helpless due to stock-outs of commodities and drugs [43, 46]. Frustration with the system have resulted in recurring healthcare worker strikes to demand for better wages and working conditions in Kenya [47]. Human-resource-neutral strategies, such as improved salaries, merit, appreciation, and opportunities for promotion need to be explored to address absenteeism [48].

Our study underscored that workload is not *consistently* high and there was an under-utilization of skilled healthcare workers on the majority of days, even under scenario [2], which suggests that current staffing numbers in small dispensaries should have sufficient time to deliver full ANC services, including integrated point-of-care testing. Low utilization has been found elsewhere: case studies from Tanzania and Chad found that only 55–60% of staff time was spent on productive activities [30]; worse has been reported in Cameroon where reproductive health staff spend only 27% on service provision [28]. Investments in improving performance of the existing workforce has potential to improve the quality of care. Motivation and performance have several determinants, but in general, salaries, prestige, work conditions, frequent high-quality supervision with audit feedback and multifaceted interventions have strong evidence to support their contribution to towards better healthcare worker performance [46].

The domain of this study was operational and its aim was to develop insight to the local implementation conditions of integrated POCT [49]. The generalizability of our findings to other settings may be limited as we only studied a single site. The study may also be weakened by the Hawthorne effect: having data collectors present at the facilities may alter healthcare worker’s behaviour. We also only collected data for 1 month and this may not be entirely representative of the operational environment over time. We focused only on women who visited the facility for MCH/PMTCT purposes and did not quantify wait times for OP or ART clients. While we have collected detailed data for MCH and PMTCT activities, we made assumptions for the duration of OP and ART consultations undergone by non-MCH women. We used an average OP consult time of 6.5 min which was similar to 7 min found in Nigeria [50] and slightly longer than 5.3 min found in Mozambique [51]. Another study found ART clients spend an average of 21.8 min on services which included time spent in registration, with CO and at the pharmacy [36]. Thus, our estimate for consult time of 10 min with the CO seems reasonable. These findings need to be reviewed with frontline healthcare workers and stakeholders to better interpret and understand their implications so that suitable strategies can be devised to adequately address these operational challenges.

## Conclusion

Using discrete-event simulation modelling with detailed facility-level data, we quantified operational outcomes of wait times, length-of-stay and nurse utilization after integrating syphilis, malaria and anaemia point-of-care testing with HIV testing during ANC consultations. We showed that nurse utilization rates fluctuated: nurses experienced spikes in workload on some days but were under-utilized on the majority of days. Overall, our model suggests there was enough time to provide adequate services and integrated testing for ANC first and re-visits with the current number of healthcare staff. While this would increase wait times and length-of-stay for a portion of women, it would significantly improve the quality of care through ensuring pregnant women receive essential antenatal services and counselling. Human-resource-neutral strategies to reduce healthcare worker absenteeism, especially on busy days, and improve their motivation and performance should be explored to ensure limited resources are used efficiently without over-stretching the system.

## Data Availability

The datasets used and/or analysed during the current study are available from the corresponding author on reasonable request.

## Abbreviations

ANC: Antenatal care
ART: Anti-retroviral treatment
CDC: Centers for disease control and prevention
CO: Clinical officer
DES: Discrete-event simulation
HDSS: Health and demographic surveillance system
HTC: HIV testing counsellor
KEMRI: Kenya Medical Research Institute
MCH: Maternal and child health
OP: Out-patient
PMTCT: Prevention of mother-to-child transmission
POCT: Point-of-care testing
POCTs: Point-of-care tests
SSA: Sub-Saharan Africa
